# Soy Protein Isolate Interacted with Acrylamide to Reduce the Release of Acrylamide in the In Vitro Digestion Model

**DOI:** 10.3390/foods12061136

**Published:** 2023-03-08

**Authors:** Yu Shen, Mengling Lv, Zhenyue Tang, Wei Liu, Yusong Zhang, Fei Teng, Xu Wang, Meili Shao, Yujun Jiang

**Affiliations:** 1 Department of Food Science, Northeast Agricultural University, Harbin 150038, China; 2 Key Laboratory of Dairy Science, Ministry of Education, Harbin 150030, China; 3 Harbin Customs Technology Center, Harbin 150030, China

**Keywords:** soy protein isolate, acrylamide, interaction mechanism, gastrointestinal digestion simulation

## Abstract

Acrylamide (AA), a common carcinogen, has been found in many dietary products.. This study aimed to explore the interaction of soybean protein isolate (SPI) with AA and further research the different effects of SPI on the AA release due to interactions in the in vitro digestion model. Analysis of variance was used to analyze the data. The results suggested that AA could bind with SPI in vitro, leading to the variation in SPI structure. The intrinsic fluorescence of SPI was quenched by AA via static quenching. The non-covalent (van der Waals forces and hydrogen bonding) and covalent bonds were the main interaction forces between SPI and AA. Furthermore, the release of AA significantly decreased due to its interaction with SPI under simulated gastrointestinal conditions. SPI had different effects on the AA release rate after different treatments. The thermal (80, 85, 90, and 95 °C for either 10 or 20 min) and ultrasound (200, 300, and 400 W for either 15, 30, or 60 min) treatments of SPI were useful in reducing the release of AA. However, the high pressure-homogenized (30, 60, 90, and 120 MPa once, twice, or thrice) treatments of SPI were unfavorable for reducing the release of AA.

## 1. Introduction

Acrylamide (AA), which is a colorless crystalline solid, can directly pass into the body via food intake, respiration, and dermal contact [1]. The relationship between dietary acrylamide and the risk of several common cancers has been widely reported. In 2002, studies revealed that high levels of AA were found in some foodstuffs fried or baked at high temperatures [2]. Relevant toxicological studies indicated that AA had an adverse impact on human health, including neurotoxicity, genotoxicity, carcinogenicity, and reproductive toxicity [3]. The health hazards of AA-contaminated food cannot be neglected.

At present, AA toxicity in food is mainly controlled by decreasing its formation and reducing its adsorption in vivo. Many methods are used to reduce the generation of AA in food, mainly by modifying raw materials, adding exogenous additives, and optimizing processing [4,5,6]. For example, researchers found that using quinoa flour instead of wheat flour could significantly reduce AA formation in biscuits compared with the control sample [7]. Furthermore, Sobol et al. reported that water immersion semi-products effectively reduced the AA content in French fries. However, the results also indicated that the exposure of potato tubers to ultraviolet-C radiation led to an increase in AA content [8]. Therefore, the human body cannot altogether avoid the intake of AA.

As is well known, only a portion of the total intake of harmful compounds is made available for subsequent absorption in the intestines [9]. Therefore, reducing the AA release could radically decrease the AA absorption in vivo. Previous studies confirmed that chicken egg albumin could be combined with AA to reduce the adsorption of AA in the intestines [10]. However, numerous gaps exist in the studies exploring the interaction of the protein with AA and the impact of food proteins on the AA release in the gastrointestinal tract due to both interactions.

In recent years, soy-based products, such as soymilk, soybean curd, and fermented soybean paste, have been appreciated by people for their nutritional value and delicious taste [11,12]. Soy protein is an important component of soy-based products, which is widely studied and applied in the food industry [13]. Studies have suggested that soy proteins can interact with multiple small molecules, such as flavonoids and phenolic acid, due to their higher biological activities [14,15]. Moreover, the structural and functional properties of soybean protein isolate (SPI) change after thermal treatment, high-pressure homogenization (HPH), and ultrasonic treatments, affecting the interactions between SPI and small molecules [16]. These interactions further influence the degree of small-molecule release and absorption in the gastrointestinal tract [17,18].

The objective of this study was to first explore the interaction of SPI with AA in terms of binding rate, Fourier transform–infrared spectroscopy (FTIR), fluorescence spectroscopy, and sulfhydryl (–SH) content. Based on the interaction between SPI and AA, the effect of nontreated and differently treated SPI on the AA release was further investigated in the in vitro digestion model. The different treatments of SPI included the thermal treatment (80, 85, 90, and 95 °C for either 10 or 20 min), HPH treatment (30, 60, 90, and 120 MPa once, twice, or thrice), and ultrasound treatment (200, 300, and 400 W for either 15, 30, or 60 min). The results of this study might help improve food quality and form the basis for seeking an effective approach to reducing AA health risks.

## 2. Materials and Methods

### 2.1. Materials

Degreased soybean flours and AA standards were bought from Yu Wang Co. (Dezhou, China) and Sigma–Aldrich Co. (St. Louis, MO, USA), respectively. The remaining reagents were purchased from Solarbio Co. (Beijing, China) unless stated otherwise.

### 2.2. Preparation of SPI

SPI was prepared by the method reported by Shen et al. [6]. Soybean solution (pH = 8.5) was centrifuged at 10,000× *g* for 20 min. The supernatant was harvested, and the pH was adjusted to 4.5. The SPI precipitate was obtained via centrifuging at 10,000× *g* for 5 min. The precipitated protein was washed several times. The SPI extracts were lyophilized to obtain the SPI powder. The protein content of SPI was determined as 93.75% using the Kjeldahl method, following the Association of Official Agricultural Chemists (AOAC) procedure No.991.20 [18].

### 2.3. Preparation of SPI–AA Complexes

AA was mixed with SPI for the interaction assay. Referring to the daily ingestion and previous literature [10,17,19], AA was used with the final concentrations of 0.5, 1, 2.5, and 5 mmol/L, and SPI with the final concentrations of 0.1%, 0.25%, 0.5%, 1%, and 2% (*w*/*v*). The interaction process was conducted in the dark for 2 h.

### 2.4. AA Quantitation Using High-Performance Liquid Chromatography

According to the methods of the AOAC procedure No.1,2005 and our previous relevant studies, an LC-20A high-performance liquid chromatography (HPLC) system (Shimadzu, Kyoto, Japan) was used to perform AA quantitation [20,21]. The flow velocity was 1 mL/min using isocratic elution. The mobile phase comprised water and methanol mixed in a 95:5 (*v*/*v*) ratio. The detection was performed using the 205 nm wavelength. The injection volume and temperature were 20 μL and 30 °C, respectively.

### 2.5. Determining the AA Binding Rate

The SPI–AA complexes were precipitated via isopropanol based on the approach discussed in the study by Schabacker et al. [10]. The quantitation of free AA in the supernatant was performed via HPLC (Section 2.4) using the following equation:

(1) $$B\left(\%\right)=\frac{A-A_{0}}{A}\times 100$$

where *B* is the AA binding rate; *A* is the total AA content; and *A*_0_ is the content of the free AA-containing supernatant.

### 2.6. FTIR Spectroscopy

The structural changes in SPI before and after interaction with AA were determined using FTIR spectroscopy (Nicolet 6700; Thermo Fisher Scientific, Waltham, MA, USA). The sample was lyophilized and ground with KBr powder in a ratio of 1:100. Afterward, the obtained mixture was pressed into a disk. The FTIR spectrum was registered at a range of 4500–500 cm^−1^ [22].

### 2.7. Fluorescence Spectroscopy

Fluorescence measurement was performed using a fluorescence spectrophotometer (F4500; Hitachi, Japan). The SPI–AA complexes were obtained as described in Section 2.3. The final SPI concentration was 0.05%, and the concentrations of AA in the mixtures were 0, 20, 40, 60, 80, 100, 120, 160, and 200 μmol/L. The spectra were determined at 297, 304, and 311 K with an emission wavelength of 300–500 nm and an excitation wavelength of 280 nm. The fluorescence intensity of the background was subtracted from the raw spectra of the complexes [23]. The following equation (Stern–Volmer) was used to analyze the fluorescence quenching:

(2) $$\frac{F_{0}}{F}=1+K_{q}\tau_{0}[Q]=1+K_{SV}[Q]$$

where *F*_0_ and *F* represent the fluorescence intensities of SPI in the presence and absence of AA. *K*_q_ and *K*_sv_ are the quenching rate constant and Stern–Volmer quenching constant, respectively. [*Q*] and *τ*_0_ represent the AA concentration and fluorophore average lifetime, respectively [24].

Previous studies indicated that the quenching mechanism was static when the *K*_q_ value was greater than 2.0 × 10^10^ L·mol^−1^·s^−1^ [24]. The following equation (double logarithmic) was used to analyze the binding situation:

(3) $$Log\frac{F_{0}-F}{F}=LogK_{A}+nLog[Q]$$

where *F*_0_, *F*, and [*Q*] are the same variables as in Equation (3). *K_A_* and *n* represent the binding constant and number of binding sites, respectively.

### 2.8. Determining the –SH Content

The SPI–AA complexes were appropriately diluted using Tris-glycine buffer by the method described by Sui et al. [25]. Then, the resulting complexes underwent reactions for 15 min upon adding the Ellman reagent. The absorbance of samples was determined at 412 nm using a spectrophotometer (UV-2401PC; Shimadzu, Japan). The –SH content was determined using the following equation:

(4) $$SH/(\mu \mathrm{mol}/g)=\frac{73.53\times A_{412\mathrm{nm}}\times D}{C}$$

where *A*_412nm_ represents the absorption of 412 nm; and *D* and *C* indicate the dilution factor and the sample concentration, respectively.

### 2.9. Effect of SPI on the Release of AA in the In Vitro Digestion Model

The effects of different concentrations and processing treatments of SPI on AA release were evaluated via the gastrointestinal digestion simulation system in vitro.

#### 2.9.1. Processing Methods of SPI

Three processing methods of SPI were used to determine whether the SPI under different treatments could affect the AA release rate. The specific treatments were performed as follows:

1. In the thermal treatment, SPI was heated at 80, 85, 90, and 95 °C either 10 or 20 min;
2. In the HPH treatment, SPI was processed at pressures of 30, 60, 90, and 120 MPa once, twice, or thrice;
3. In the ultrasonic treatment, the SPI was treated at 200, 300, and 400 W for either 15, 30, or 60 min following the method described by Hu et al. [26]. The sample temperature was maintained at 25 °C–35 °C throughout the process.

#### 2.9.2. Preparing the SPI–AA Mixture

The SPI of nontreatment, thermal treatment, HPH treatment, and ultrasound treatment was mixed with AA at a certain ratio to prepare SPI–AA mixtures. The final concentration of SPI was 1%, 3%, and 5% (*w*/*v*). AA was added at a final concentration of 0.5 and 2.5 mmol/L. Then, the mixture was used in the in vitro gastrointestinal digestion simulation system.

#### 2.9.3. Simulating Gastrointestinal Digestion In Vitro

The simulated salivary fluid (SSF), simulated gastric fluid (SGF), and simulated intestinal fluid (SIF) were produced using appropriate amounts of enzymes, electrolyte stock solutions (Table 1), and calcium dichloride (CaCl_2_) [27]. The experiments conducted were as follows:

1. In the oral phase, 10 mL of the SPI–AA mixture in each group was mixed with α-amylase (1 mL, 1500 U/mL), SSF electrolyte (8 mL), CaCl_2_ (50 μL, 0.3 mol/L), and water (1.95 mL). The resultant mixture was cultured at 55 rpm and 37 °C for 2 min;
2. In the gastric phase, the samples after oral digestion were mixed with the SGF electrolyte in a certain proportion. Pepsin was added at a final concentration of 2000 U/mL. The pH of the mixture was adjusted to 3.0. The resultant mixture was cultured at 55 rpm and 37 °C for 2 h.

In the intestinal phase, the samples after oral and gastric digestion were mixed with the SIF electrolyte in a certain proportion. Pancreatin and bile were added at a final concentration of 2000 U/mL and 0.16 mol/L, respectively. The pH of the mixture was adjusted to 7.0. The resultant mixture was cultured at 55 rpm and 37 °C for 2 h.

The samples of the gastric and intestinal phases were centrifuged at the end of the in vitro digestion process, and the supernatants were used for the quantitative analysis of AA using HPLC (Section 2.4).

The following equation was used for calculating the release of AA [28]:

(5) $$R(\%)=\frac{A_{1}}{A}\times 100$$

where *R* is the AA release rate. *A*_1_ and *A* are the contents of free and total AA in the simulated digestive fluid of different phases, respectively.

### 2.10. Data Analysis

The assays were performed in triplicate, and the data were presented as mean ± standard deviation. The data were analyzed for homogeneity of variance and normal distribution using Levene’s test and the Kolmogorov–Smirnov (K-S) test, respectively. The Analysis of variance was performed to assess the data using SPSS 23.0 (IBM SPSS Inc., Chicago, IL, USA). The significance level for analyses was set at α = 0.05 (*p* < 0.05). Origin 2017 software was used to generate the graphs.

## 3. Results and Discussion

### 3.1. In Vitro Interaction of SPI with AA

**Figure 1:**
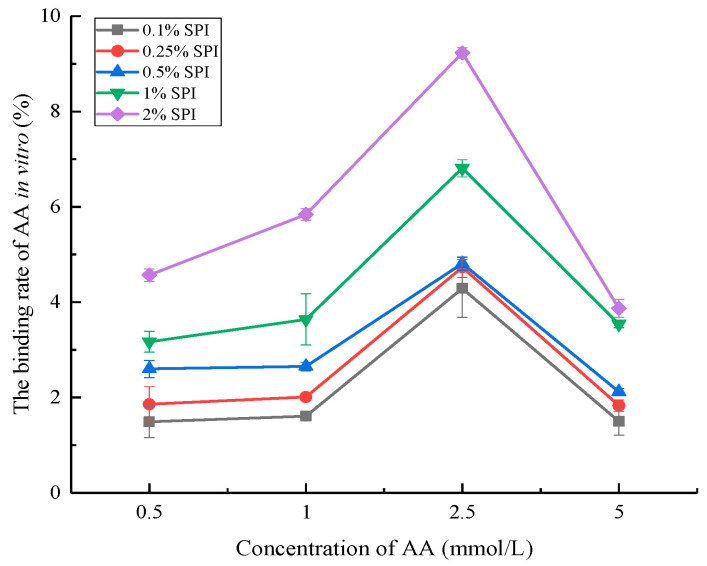
The binding rate of AA in vitro under different SPI concentrations. The amounts of 0.1% SPI, 0.25% SPI, 0.5% SPI, 1%, and 2% SPI represented the concentration of SPI (error bars represent the mean ± SD of triplicate assays).

The binding rate of AA changed with the increase in the concentrations of SPI and AA (Figure 1), suggesting that AA could interact with SPI in vitro. Similar results were reported by Schabacker et al. [10], who found that chicken egg albumin was capable of interacting with AA during cooking and in intestinal conditions. Moreover, related studies suggested that SPI could bind to small molecule substances (e.g., polyphenols and tangeretin) in vitro, and the binding rate was closely associated with the SPI concentration and bound substances [29,30].

The results of this study indicated an increase in the AA binding rate with the increase in the SPI concentration (from 0.1% to 2%). This was consistent with the findings by Xiang et al. [31], suggesting that SPI binding to curcumin increased with the enhancement in the SPI concentration due to the increase in the number of binding sites. Moreover, the AA binding rate increased first and then decreased with the increase in the AA concentration (from 0.5 to 5 mmol/L). The AA binding rate reached the maximum at the AA concentration of 2.5 mmol/L. The results suggested that the amount of bound AA increased with increasing AA concentration. However, the increase in the amount of bound AA was smaller than the increase in the amount of total AA. In turn, the binding rate of AA showed a decreasing trend at the AA concentration of more than 2.5 mmol/L due to the substantial increase in the total AA amount [32]. Based on previous studies, we speculated that the interaction between SPI with AA increased and approached saturation with the increase in the AA concentration [33,34].

### 3.2. FTIR Analysis

**Figure 2:**
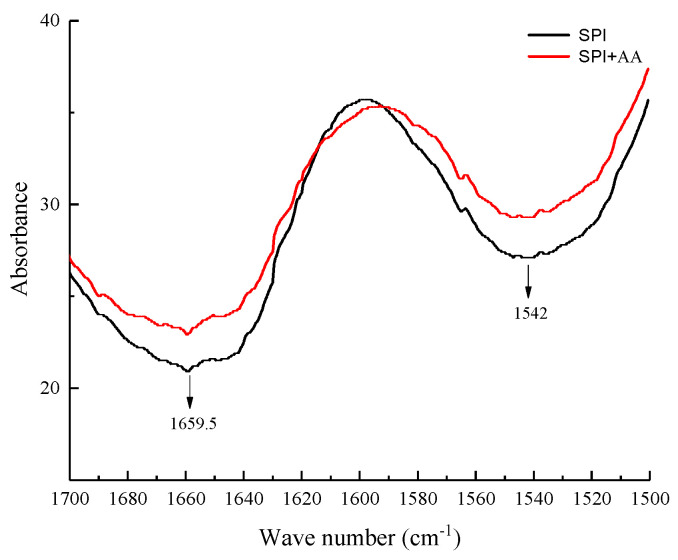
The FTIR spectra of SPI and SPI-AA complexes.

The FTIR analysis was performed to determine the structural change in soy protein before and after interaction with AA. The amide I (1700–1600 cm^−1^) and II bands (1600–1500 cm^−1^) at the FTIR spectrum represented the molecular vibrations in the proteins, offering important information about the dipole–dipole interaction, hydrogen-bonding patterns, and changes in the secondary structure of the proteins [35,36]. As shown in Figure 2, the amide I and II bands of the SPI sample were at 1659.5 and 1542 cm^−1^, respectively. The peak intensity of amide I and II bands obviously changed in the SPI samples after interaction with AA, suggesting that the secondary structure of SPI changed [25,37]. The percentage of β-sheet and random coil in SPI decreased, and that of β-turn in SPI increased after SPI interacted with AA (Table 2). This finding was similar to the studies by Sui et al. [25] and Zhang et al. [38] which indicated that the secondary structure of SPI was altered after SPI interacted with anthocyanins and vitamin D_3_. Hence, we speculated that AA could interact with SPI, accompanied by the formation of chemical bonds [39].

### 3.3. Fluorescence Spectroscopy Analysis

**Figure 3:**
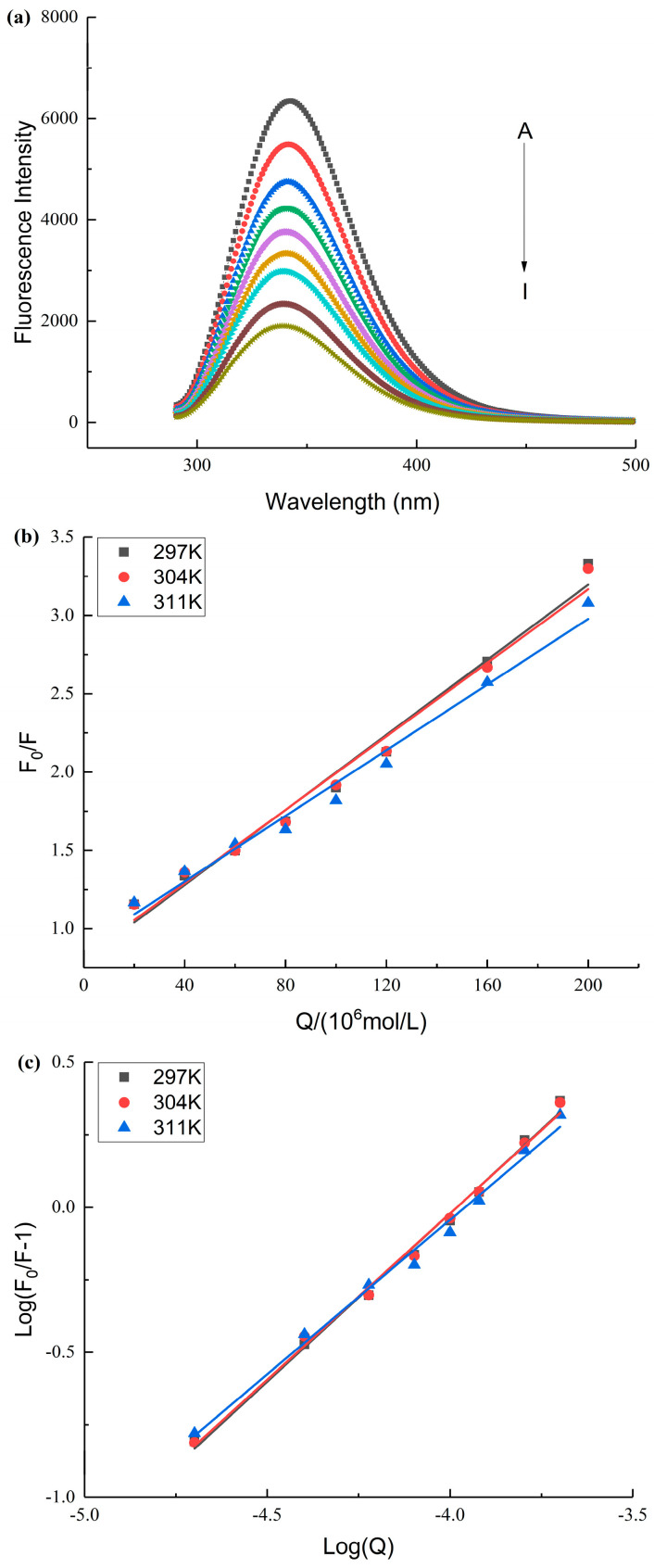
(**a**): The effect of AA on the fluorescence spectrum of SPI. A–I represents the different final concentrations of AA with 0–200 μM. (**b**): The Stern–Volmer curves of SPI and AA at different temperatures. (**c**): The double logarithm regression curve of SPI and AA at different temperatures.

To explore the interaction mechanism of SPI with AA, the fluorescence spectra of SPI after SPI interacted with different concentrations of AA were determined in this study [40,41]. The fluorescence intensity of SPI reduced with an increase in AA concentration (Figure 3a). The maximum emission wavelength (*λ*_max_) of SPI blue-shifted after SPI interacted with AA. The change in *λ*_max_ and the reduction in fluorescent intensity suggested that the microenvironment surrounding the fluorescent amino acids in SPI changed after SPI interacted with AA, leading to the reduction in the fluorescent quantum yields of SPI [14,42].

SPI fluorescence intensities were analyzed at different temperatures to determine the fluorescence quenching mechanism (dynamic and static) [43]. The *K*_sv_ values declined with an increase in temperature, and the magnitude of *K*_q_ was 10^12^ (Table 3 and Figure 3b), indicating that SPI and AA interacted via a static quenching mechanism [40,44]. The *K*_A_ values were in the order of magnitude 10^4^ (Table 3). This suggested that AA interacted strongly with SPI [40,45].

The thermodynamic parameters could be applied to analyze the main interaction forces between proteins and other molecules [23,46]. The results indicated that the main forces between the SPI and AA were hydrogen bonding and van der Waals forces (Table 3). SPI contained several –OH groups that facilitated hydrogen bonding, further verifying the interactions between SPI and AA [39]. SPI and AA have functional groups that could generate the polarized molecules, indicating the reasonable existence of van der Waals forces [47]. The Δ*G* value was negative, indicating that the interaction between SPI and AA proceeded spontaneously (Table 3) [48].

### 3.4. Analysis of the –SH Content

**Figure 4:**
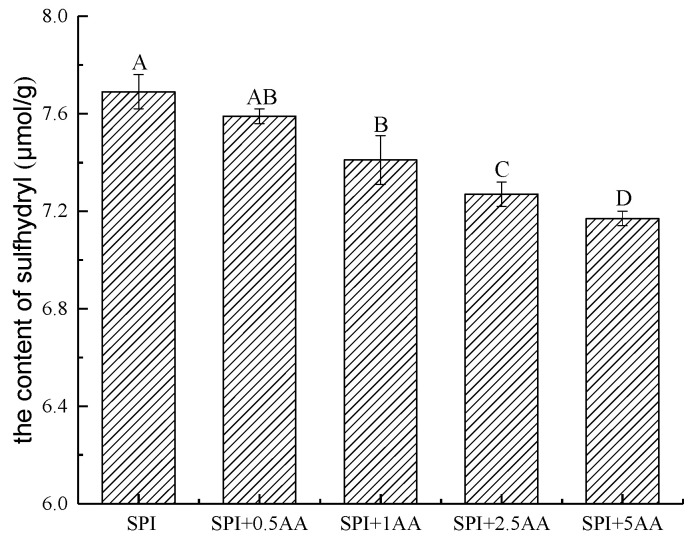
The content of –SH in the SPI and SPI-AA complexes. Significant differences between groups (*p* < 0.05) are indicated by different capital letters. 0.5 AA, 1 AA, 2.5 AA, and 5 AA represented the concentration of AA (the unit is mmol/L) (error bars represent the mean ± SD of triplicate assays).

The –SH groups in SPI participate in many interactions in food systems due to their higher chemical activity [49]. The correlation studies reported that the –SH group of thiols rapidly reacted with the carbon–carbon double bonds of AA and generated the corresponding adducts [24]. The –SH group of cysteine contributes to the scavenging reaction of AA by binding with AA [50]. Therefore, the change in the –SH content was measured in SPI before and after SPI interacted with AA to further explore whether the –SH groups in SPI were involved in the interaction. Following the result of the Levene’s and K-S tests, the parametric statistics were used to analyze the data of –SH content. As shown in Figure 4, the –SH content of SPI significantly reduced after SPI interacted with AA. The –SH content of SPI gradually decreased with an increase in AA concentration. This is consistent with the results in the previous studies, which found a decrease in the –SH content of SPI after interaction with phenolic compounds [25,40]. The correlative studies showed that proteins could eliminate AA by the sulfhydryl groups of amino acid side chains that interacted with AA [50]. Therefore, we speculated that –SH groups were involved in the interaction between SPI and AA, forming a covalent bond. Meanwhile, the interaction of AA and SPI might change the functional properties of the proteins, which requires further investigation [51].

### 3.5. Effect of SPI on the Release of AA

**Figure 5:**
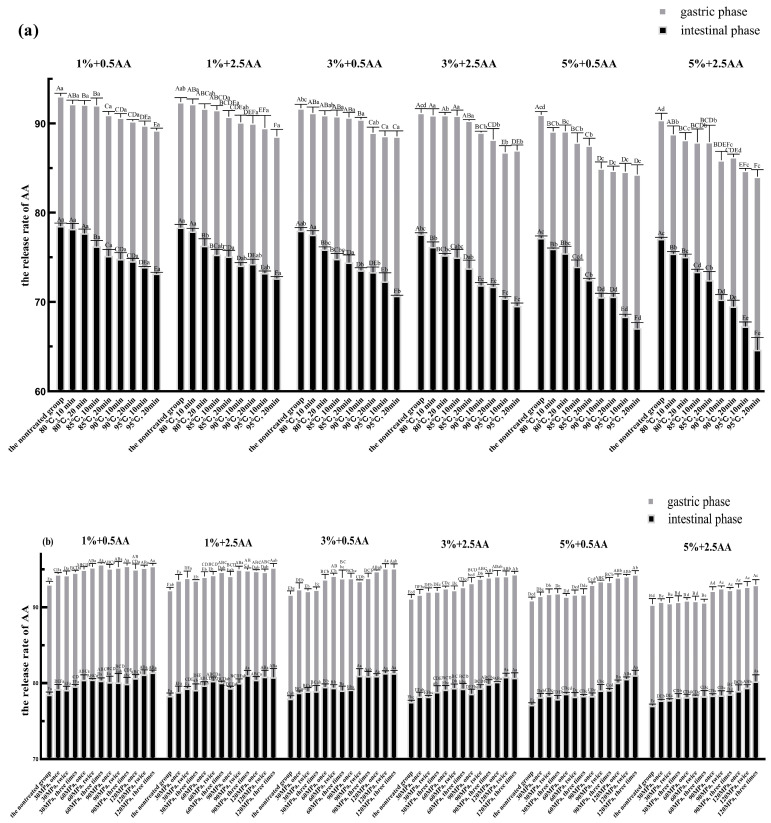

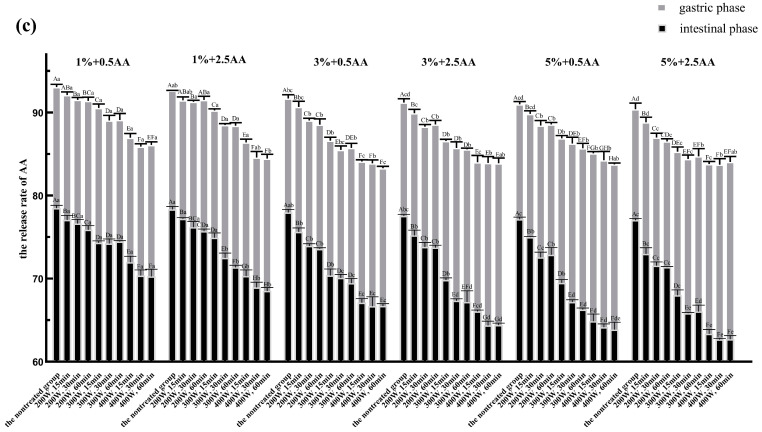
The AA release rate of nontreated, thermal-treated, HPH-treated, and ultrasound-treated groups in the gastric and intestinal phases. (**a**): Thermal treatment group; (**b**): HPH treatment group; (**c**): Ultrasound treatment group. Significant differences (*p* < 0.05) within the same experimental groups are indicated by different capital letters at the same digestive stage. Significant differences (*p* < 0.05) between groups are indicated by different small letters at the same digestive stage. 1%, 3%, and 5% represented the concentration of SPI before the in vitro digestion simulation. The figures 0.5 AA and 2.5 AA represented the concentration of AA (the unit is mmol/L) (error bars represent the mean ± SD of triplicate assays).

Based on the aforementioned studies about the interaction between SPI and AA, we further explored the effect of (nontreated, thermal-treated, HPH-treated, and ultrasound-treated) SPI on the AA release in the in vitro digestion model due to both interactions. Based on the results of Levene’s and K-S tests, the parametric statistics could be used to analyze the data of the AA release rate. The results showed that the release of AA significantly decreased after the addition of SPI in the gastrointestinal digestion environment (Figure 5). The release of AA reduced gradually with an increase in SPI concentration. The results were consistent with the interaction of SPI and AA in vitro (Section 3.1). Related research demonstrated that the release of phenolic and bioactive substances can be obviously affected by interactions between phenolic and bioactive substances with the protein [52,53]. Several studies have indicated that protein leads to a decrease in small molecule release due to intermolecular interactions [10,51,54]. Therefore, it is speculated that the reduction in AA release may be closely associated with the interaction of SPI and AA during in vitro digestion.

It is evident from Figure 5 that the AA release rates were significantly lower in the same samples under intestinal digestive conditions than in those under gastric digestive conditions. It is generally known that the intestinal tract further hydrolyzes SPI into smaller peptides and amino acids based on gastric digestion [40]. Peptides and amino acids have more nucleophilic groups compared with proteins, which will lead to more exposure of binding sites [55]. Furthermore, relevant research has reported that the simulated intestinal pH condition favored the binding of proteins with AA via the Michael addition reaction [10,55]. Hence, we speculated that the lower release rate of AA was related directly to the increase in binding sites and the pH value of physiological environment under intestinal digestive conditions. The structural and functional properties of SPI were affected by processing conditions, such as thermal, HPH, and ultrasonic treatments, when applied in soy protein processing [16]. SPI was processed at varying degrees of thermal, HPH, and ultrasonic treatments before simulated gastrointestinal digestion to further explore the effect of differently treated SPI on the release of AA. As shown in Figure 5a, the AA release rate in the thermal treatment group was significantly less than that in the nontreated group in the gastric and intestinal phases (*p* < 0.05). Moreover, the AA release rate decreased gradually with an increase in the degree of thermal treatment. The AA release rate was the lowest in all groups in the gastric and intestinal phases when SPI was heated at 95 °C for 20 min.

As shown in Figure 5b, the AA release rate in the HPH treatment group was significantly higher than that in the nontreated group in the gastric and intestinal phases (*p* < 0.05). The AA release rate increased gradually with the increase in the strength of the HPH treatment. When SPI was treated at a pressure of 120 MPa three times, the AA release rate was the highest for all groups in the gastric and intestinal phases.

The AA release rate in the ultrasonic treatment group was significantly less than that in the nontreated group in the gastric and intestinal phases (*p* < 0.05) (Figure 5c). The AA release rate decreased gradually with the increase in the strength of the ultrasonic treatment. The AA release rate reached its minimum value for all groups in the gastric and intestinal phases after SPI was treated at 400 W for 60 min.

Taken together, each of the aforementioned three SPI processing methods had different effects on the AA release rate. The AA release rate significantly decreased with the increase in the strengths of the thermal and ultrasonic treatments. This reduction might be closely associated with the thermal and ultrasonic treatments of SPI, leading to the unfolding of structure and reduction in particle size of soy protein and the exposure of some internal groups and more active sites. This could reduce the energy barrier for adsorption leading to the increase in protein’s affinity for AA and the improvement in adsorption efficiency [56,57,58]. Based on the previous studies, we speculated that another primary reason for the reduction in AA release was the increase of –SH group content in SPI after thermal or ultrasonic treatment due to the breakage of molecular chains and the destruction of disulfide bonds between molecules respectively [38,59]. However, the AA release rate increased with the increase in the strength of the HPH treatment. This was possibly due to a loosening of soy 11S globulin molecular structure during the HPH treatment, resulting in increased cross-linking of the –SH group and the formation of disulfide bonds [60,61]. This further reduced the content of the –SH group, which ultimately led to an increase in the AA release rate in the HPH treatment group. Through HPH treatment, SPI is presumably more prone to aggregation due to the denaturation, leading to the precipitation and flocculation of SPI. This will reduce the interaction of SPI with AA, and then increase the release of AA [62].

## 4. Summary

In this study, the interaction of SPI with AA was found via the change in the AA binding rate, which changed with the increase in the concentration of SPI and AA. Fluorescence spectra further revealed that the quenching of AA to SPI was static, and the interaction reaction proceeded spontaneously due to the negative Δ*G*. Meanwhile, with the comprehensive analysis of the results of thermodynamic parameters and –SH content in SPI, it was found that the interaction of SPI with AA occurred mainly via van der Waals forces, hydrogen bonding, and covalent bonding. Furthermore, the release of AA significantly decreased after the addition of (nontreated, thermal-treated, HPH-treated, and ultrasound-treated) SPI in the in vitro digestion model, which further demonstrated the interaction between SPI with AA under gastrointestinal digestion conditions. In addition, SPI after different treatments had different effects on the AA release rate. The thermal (80, 85, 90, and 95 °C for either 10 or 20 min) and ultrasound treatments (200, 300, and 400 W for either 15, 30, or 60 min) of SPI contributed to the decrease in the AA release rate compared with the nontreated group. However, the HPH (30, 60, 90, and 120 MPa once, twice, or thrice) treatment of SPI was not conducive to the AA release compared with the nontreated group. The findings of this study might help improve food quality and establish the basis for seeking an effective approach to reducing the absorption of AA in the body.

## Figures and Tables

**Table 1:** Preparation of SSF, SGF, and SIF electrolytes.

		SSF Electrolyte		SGF Electrolyte		SIF Electrolyte	
		pH = 7		pH = 3		pH = 7	
Content	Concentration (mol/L)	Volume (mL)	Final Concentration (mmol/L)	Volume (mL)	Final Concentration (mmol/L)	Volume (mL)	Final Concentration (mmol/L)
KCl	0.5	15.1	15.1	6.9	6.9	6.8	6.8
KH_2_PO_4_	0.5	3.7	3.7	0.9	0.9	0.8	0.8
NaHCO_3_	1	6.8	13.6	12.5	25	42.5	85
NaCl	2	--	--	11.8	47.2	9.6	38.4
MgCl_2_(H_2_O)_6_	0.15	0.5	0.15	0.4	0.1	1.1	0.33
(NH_4_)_2_CO_3_	0.5	0.06	0.06	0.5	0.5	--	--
HCl	6	0.09	1.1	1.3	15.6	0.7	8.4

Note: The final volume was made up to 500 mL with distilled water.

**Table 2:** Secondary structure percentages of SPI and SPI-AA based FTIR analysis. Reprinted with permission from Ref. [6]. Copyright ©2021, John Wiley and Sons.

	β-Sheet	Random Coil	α-Helix	β-Turn
SPI	40.17%	16.78%	17.29%	25.77%
SPI + AA	33.01%	8.82%	17.62%	40.54%

**Table 3:** Fluorescence quenching parameters for SPI and SPI-AA.

T/K	297 K	304 K	311 K
K_SV_/(L·mol^−1^)	1.20 × 10^4^	1.17 × 10^4^	1.05 × 10^4^
K_q_/(L·mol^−1^·s^−1^)	1.20 × 10^12^	1.17 × 10^12^	1.05 × 10^12^
R^2^ (S-V)	0.9835	0.9848	0.9833
K_A_/(L·mol^−1^)	4.13 × 10^4^	3.68 × 10^4^	1.65 × 10^4^
n	1.16	1.15	1.07
R^2^ (double logarithm)	0.9951	0.9948	0.9906
ΔH/(kJ·mol^−1^)	−50.17	−50.17	−50.17
ΔG (kJ·mol^−1^)	−26.25	−26.57	−25.11
ΔS/(J·mol^−1^·K^−1^)	−80.56	−77.64	−80.60

## Data Availability

Not applicable.
